# Knowledge, attitude, and practices toward dengue fever among the public: a cross-sectional study in the Western region of Saudi Arabia

**DOI:** 10.3389/fpubh.2024.1327427

**Published:** 2024-03-06

**Authors:** Munerah Hamed

**Affiliations:** Department of Pathology, Faculty of Medicine, Umm Al-Qura University, Makkah, Saudi Arabia

**Keywords:** dengue fever, knowledge, attitude, practices, Western region, Saudi Arabia

## Abstract

**Background:**

Dengue fever (DF) is a primary viral disease transmitted to humans by mosquitoes, imposing a significant economic and health burden in numerous regions globally. There is a lack of studies addressing the importance of health education regarding DF in Saudi Arabia. Therefore, this study aimed to assess predictors influencing knowledge, attitude, and practices of the Western region public in Saudi Arabia related to DF.

**Methods:**

This cross-sectional study was conducted as an online self-administered questionnaire collected from July 2023 until August 2023, included 695 participants from the Western region residents aged ≥18 years. Data collected involved sociodemographics, participants’ knowledge, attitude, and practices toward DF. We presented the descriptive data as mean ± standard deviations (SD) and medians [interquartile range (IQR)] for continuous variables, while categorical variables were presented as frequencies [percent (%)].

**Results:**

Good participants’ knowledge and attitude toward DF were observed among participants [median score 21.0 (interquartile range 16.0–24.0)] out of 35 and [median score 4.00 (interquartile range 3.00–5.00)] out of 5, respectively. Limited practices toward DF were observed among participants [median score 3.0 (interquartile range 0.00–5.00)] out of 8. Participants’ knowledge and attitude were not correlated. Participants’ education level and employment status predicted participant’s knowledge of DF. Participants’ age, monthly income, employment status and marital status predicted participants’ attitude toward DF. Participants’ age, marital status, education levels and income predicted participants’ practices toward DF.

**Conclusion:**

Knowledge, attitude, and practices toward DF among adults in Saudi Arabia can be predicted by specific sociodemographic characteristics. Implementing interferences that focus on improving public practices toward DF is imperative.

## 1 Introduction

Dengue fever (DF) is a significant public health problem worldwide, caused by the dengue virus. It is one of the human epidemic arboviruses transmitted to humans by Aedes mosquitoes (1–3). In addition, human transmission of dengue is currently endemic in more than 125 countries, including Saudi Arabia (4). Specifically, the Western region (WR) of Saudi Arabia is at risk and burden of dengue (5, 6). A previous study estimated the prevalence of IgG antibodies against dengue in Makkah, Jeddah and Madinah cities’ population in Saudi Arabia showed that one in four residents is seropositive. More importantly, in some districts of these cities, the seropositive rates have reached more than 50% of the population in those districts (7). These studies imply the possibility of an increase in the prevalence of DF in Saudi Arabia. In addition, a previous meta-analysis study revealed that both high-temperature and humidity are associated with the risk of dengue virus infection (8). Therefore, DF is prevalent among the Western coastline of Saudi Arabia as it provides favorable conditions for the transmission of dengue infection, characterized by a hot, humid, and rainy climate, as well as the formation of stagnant water following heavy rainfall (9). The Western region of Saudi Arabia has experienced a series of substantial rainfall events between 2017 and 2023, resulting in a rapidly advancing DF epidemic in the area.

Dengue virus infection can lead to a broad range of clinical presentations, varying from a mild flu-like illness known as DF to a condition that pose risk to life known as dengue shock syndrome. Dengue fever symptoms typically involve fever, rash, nausea, vomiting, and body aches. At the same time, the DF complication, dengue shock syndrome, is characterized by severe blood vessels bleeding and shock, with mortality rates reaching as high as 20% if remains untreated (10). The previous classification system by the World Health Organization categorized dengue disease into three classifications: undifferentiated fever, dengue fever, and dengue hemorrhagic fever. The latter was further subdivided into four levels of severity, where levels III and IV representing dengue shock syndrome, which is a life-threatening stage of the disease. In 2009, the World Health Organization modified the DF classification, as a replacement for the previous categories into; (1) severe dengue (2), dengue with warning signs, and (3) dengue without warning signs (11).

Luckily, DF is a preventable disease that can be controlled via community-based, biological, or chemical control programs (12). The general population’s awareness, beliefs, and behaviors, collectively referred to as knowledge, attitudes, and practices (KAP), play a crucial role in preventing dengue virus infection (13, 14). It has been demonstrated that a lack of knowledge regarding dengue transmission and preventive measures can contribute to the increased risk of DF transmission (14), emphasizing the importance of expanding knowledge on DF prevention to control dengue epidemics (15). Therefore, prompt and appropriate medical care provided by experienced healthcare professionals is critical in effectively treating DF, as it helps prevent complications and reduces the fatality rate (16, 17).

Although the occurrence of DF has been on the rise in the WR of Saudi Arabia (18), there is a lack of published research documenting these outbreaks and examining the associated risk factors, as well as the KAP of the community. Therefore, this study attempts to investigate KAP within the WR of Saudi Arabia community. The findings from this study aimed to provide valuable insights into identifying existing gaps and enhancing disease control and prevention measures.

## 2 Materials and methods

### 2.1 Study population

In our cross-sectional study, we incorporated a total of 659 people of the WR of Saudi Arabia aged ≥18 years. Exclusion criteria are lack of knowledge concerning DF and missing data. The study required a minimum participants count of 239 people, using the Epi Info TM (Epi Info v5.5.11, CDC, Atlanta) with a 95% confidence level, expected prevalence of practicing individuals with knowledge is 40%, whereas proportion of participants who practice with limited knowledge is 10%.

### 2.2 Data collection

Data from our cross-sectional study were obtained through an online survey distributed via a social media application (WhatsApp). The survey invitation link was sent to contacts who are ≥18 years old and reside in the WR of Saudi Arabia. Snowball sampling technique was used to recruit initially invited participants to share the link with others who meet the inclusion criteria.

The survey collected sociodemographic data [Province of residency (Makkah province = 1 and Madinah province = 2), age (18–30 years = 1; 31–50 years = 2; > 50 years = 3), gender (male = 1; female = 2), marital status (married = 1; single = 0), education level (< high-school = 1; high-school = 2; diploma = 3; bachelor’s degree = 4; postgraduate = 5), employment status (unemployed = 0; student = 1; employed in non-health sector = 2; employed in health sector = 3), monthly income in Saudi Riyal (SR) (≤ SR 5,000 = 1; SR 5,001–10,000 = 2; SR 10,001–15,000 = 3; SR 15,001–20,000 = 4; > SR 20,000 = 5)]. The survey questionnaire was translated into Arabic. Then, expert and content validity were conducted by two experts in the field. Additionally, a pilot testing was carried out to ensure the suitability and the comprehension of items among the population of Saudi Arabia. All participants provided their consent prior to participating in the study. This study received ethical approval from the Biomedical Research Ethics Committee at Umm Al-Qura University [No. HAPO-02-K-012-2023-06-1642]. Data pertaining to the KAP of the WR population of Saudi Arabia regarding DF were collected via questions adapted from a study carried out within Middle Eastern/Arabic-speaking communities (19).

### 2.3 Assessment of the knowledge of the public in Saudi Arabia’s Western region regarding dengue fever

This section included a total number of 35 items with a total score of 35 points. Questions were as follows: (1) Have you heard about DF?; (2) Do symptoms of DF include fever?; (3) Do symptoms of DF include rash?; (4) Do symptoms of DF include muscle and joint pain?; (5) Do symptoms of DF include severe headache?; (6) Do symptoms of DF include nausea and vomiting?; (7) Do symptoms of DF include pain behind the eyes?; (8) Do complications of DF include hepatitis?; (9) Do complications of DF include neurological disorders?; (10) Do complications of DF include blood vessels damage?; (11) Do complications of DF include bleeding and hypotension?; (12) Do complications of DF include organ failure?; (13) Do complications of DF include death?; (14) Do modes of transmission include mosquito bites?; (15) Do modes of transmission include direct contact with an infected person?; (16) Do modes of transmission include eating polluted food?; (17) Do modes of transmission include drinking polluted water?; (18) Transmission is not possible?; (19) Is DF contagious?; (20) Is the peak of mosquito bites in the early mornings?; (21) Is the peak of mosquito bites in the evening before sunset?; (22) Is the peak of mosquito bites in the afternoon?; (23) Is the peak of mosquito bites at night?; (24) Can you avoid DF transmission?; (25) Do factors that increase mosquitos include stagnant water?; (26) Do factors that increase mosquitos include uncovered water containers?; (27) Do factors that increase mosquitos include poor ventilation and dark, damp areas?; (28) Is eliminating stagnant water a measure to avoid mosquitos?; (29) Is using airborne insecticide a measure to avoid mosquitos?; (30) Is covering the body a measure to avoid mosquitos?; (31) Is using Mosquito-repellent body cream/spray a measure to avoid mosquitos?; (32) Is using window/door screen a measure to avoid mosquitos?; (33) Is using fans a measure to avoid mosquitos?; (34) Is covering water containers a measure to avoid mosquitos? and (35) Is using electric bug zappers a measure to avoid mosquitos?. Responses to items 1–35 are shown in Table 1. Correct responses were scored as “1” and other responses were scored as “0”. Internal reliability of the tool used to assess the knowledge of DF was high among our study population (Cronbach’s Alpha = 0.86).

**Table 1:** Knowledge, attitude, and practices of participants toward dengue fever (DF) in the Western region of Saudi Arabia (*n* = 616).

Variable		*n*	%
Knowledge toward DF			
1	Heard of DF. Yes	616	100.0
Symptoms of DF			
2	Fever. Yes	549	89.1
3	Rash. Yes	180	29.2
4	Muscle and joint pain. Yes	396	64.3
5	Severe headache. Yes	404	65.6
6	Nausea and vomiting. Yes	298	48.4
7	Pain behind the eyes. Yes	109	17.7
	IDK	43	7.0
Complications of DF			
8	Hepatitis. Yes	89	14.4
9	Neurological disorders. Yes	71	11.5
10	Blood vessels damage. Yes	95	15.4
11	Bleeding or hypotension. Yes	163	26.5
12	Organ failure. Yes	155	25.2
13	Death. Yes	202	32.8
	IDK	271	44.0
Mode of transmission			
14	Mosquito bite. Yes (correct response)	550	89.3
15	Direct contact with an infected person. No	552	89.6
16	Eating polluted food. No	558	90.6
17	Drinking polluted water. No	491	79.7
18	Infection is impossible. No	611	99.2
	IDK	40	6.5
19	Is DF Contagious? No	206	33.4
Peak of mosquito bite			
20	Early morning. Yes (correct response)	132	21.4
21	Evening before sunset. Yes (correct response)	188	30.5
22	Afternoon. No	40	6.5
23	At night. No	169	27.4
	IDK	284	46.1
24	Can You Avoid Dengue Infection? Yes	529	85.9
Factors that increase mosquitos			
25	Stagnant water. Yes	593	96.3
26	Uncovered water containers. Yes	438	71.1
27	Poor ventilation and dark, damp areas. Yes	236	38.3
	IDK	23	3.7
Do you know the preventive measures to avoid DF			
28	Avoid stagnant water. Yes	576	93.5
39	Use airborne insecticide. Yes	532	86.4
30	Cover the body. Yes	491	79.7
31	Mosquito-repellent body cream/spray. Yes	432	70.1
32	Use window/door screen. Yes	370	60.1
33	Use fans. Yes	300	48.7
34	Cover water containers. Yes	233	37.8
35	Electric bug zapper. Yes	177	28.7
	IDK	41	6.7
Attitude toward DF			
1	Think that DF symptoms are similar to those of COVID-19		
	No/IDK	308	50.0
	Yes	308	50.0
2	Think that DF is dangerous		
	No/IDK	131	21.3
	Yes	485	78.7
3	Think society should work together to prevent DF		
	No/IDK	27	4.4
	Yes	589	95.6
4	Willing to take preventive measures		
	No	50	8.1
	Yes	566	91.9
5	Feel responsible for reporting stagnant water		
	No	220	35.7
	Did not know how to report	206	33.4
	Yes	190	30.8
Practices toward DF			
1	Have you taken any preventive measures for DF		
	Yes	368	59.7
	No	248	40.3
	Types of measures taken to prevent DF		
2	Get rid of stagnant water	371	60.2
3	Use airborne insecticide	318	51.6
4	Cover the body	250	40.6
5	Use window/door screen	100	16.2
6	Use fans	176	28.6
7	Electric bug zapper	49	8.0
	None of that	117	19.0
8	Sought medical help when suspecting DF of self/relative		
	Yes	119	19.3
	No/Never infected	497	80.7

The response “IDK” was provided in all questions included in the tool.

### 2.4 Assessment of the attitude of the public in Saudi Arabia’s Western region regarding dengue fever

This section included a total number of 5 items with a total score of 7 points. Questions were as follows: (1) I think DF symptoms are similar to that of COVID-19; (2) I think that DF is dangerous; (3) I think the society should work together to prevent DF; (4) I am willing to take preventive measures to avoid DF and (5) I feel responsible for reporting stagnant water to be eliminated. We also added a further item to describe the trusted source of information, although no numerical score was assigned to this item. The options of trusted sources of information included “National governments,” “International organizations,” “Medical research,” “Internet and social media” and “Educational campaigns.” Responses to items 1–5 are shown in Table 1. Correct responses were scored as “1” and other responses were scored as “0,” except for the response “Did not know how to report” was scored as “2.”

### 2.5 Assessment of the practices of the public in Saudi Arabia’s Western region regarding dengue fever

This section included a total number of 8 items with a total score of 8 points. Questions were as follows: (1) have taken any preventive measures to avoid DF; (2) Have eliminated stagnant water; (3) Have used airborne insecticides; (4) Have covered the body; (5) Have used window/door screens; (6) Have used fans; (7) Have used electric bug zappers; and (8) Have sought medical help when suspecting infection of DF. Responses to items 1–8 are shown in Table 1, and coded as “Yes” = 1 and “No” = 0.

### 2.6 Statistical analysis

Descriptive data were presented as mean ± standard deviations (SD) and median [interquartile range (IQR)] for continuous variables, while categorical variables were presented as frequencies [percent (%)]. The Shapiro–Wilk test was employed to evaluate the normality of the distributions of all continuous variables; the distribution of KAP toward DF were skewed (*p* < 0.05, for all). Mann–Whitney and Kruskal Wallis tests were conducted to compare the median of KAP across different groups. Pairwise comparisons were performed to identify significant findings among the different groups. Spearman’s correlation test was employed to assess the correlations between participants’ KAP and DF. Simple linear regression analyses were conducted to determine if sociodemographic characteristics could predict the score for participants’ KAP toward DF. All tests were conducted as two-tailed with a 95% confidence level. Correcting for multiple testing was done by pairwise comparisons using the Bonferroni adjustment system (0.05/number of comparisons carried out in the same test). The Statistical Package for the Social Sciences was utilized for data analysis in this study (SPSS 25, SPSS Inc., Chicago, IL).

## 3 Results

### 3.1 Sociodemographic characteristics of the study sample

A total number of participants included in this analysis was 616 participants, after excluding individuals who reported that they had not heard about DF (*n* = 25, 3.8%) and participants with missing data (*n* = 18, 2.7%). Data related to the province of residence display that 83.9% (*n* = 517) of respondents included in this study were from the Makkah province of Saudi Arabia, whereas 16.1% (*n* = 99) were from Madinah province. Proportions of participants ages 18–30 years, 31–50 years, and > 50 years were 46.9% (*n* = 289), 32.8% (*n* = 202), and 20.3% (*n* = 125), respectively. 60.7% of participants were males (*n* = 374). In addition, 50.3 (*n* = 310) were single, whereas 49.7 (*n* = 306) were married. Over half of the participants hold a bachelor’s degree (59.3%, *n* = 365). 28.4%, n = 175 reported being employed in non-health sectors, while 10.9% (*n* = 67) employed in health sectors. About half of the participants (49.2%, *n* = 303) reported a monthly income of ≤ SR 5,000. The sociodemographic features of the study sample are explained in Table 2.

**Table 2:** Sociodemographic characteristics of the study sample (*n* = 616).

Characteristics	*n*	%
Province of residency		
Makkah	517	83.9
Madinah	99	16.1
Age in years		
18–30	289	46.9
31–50	202	32.8
>50	125	20.3
Gender		
Male	374	60.7
Female	242	39.3
Marital status		
Married	306	49.7
Single	310	50.3
Education level		
<High-school	12	1.9
High-school	102	16.6
Diploma	30	4.9
Bachelor	365	59.3
Postgraduate	107	17.4
Employment status		
Unemployed	163	26.5
Student	211	34.3
Employed non-health sector	175	28.4
Employed in health sector	67	10.9
Monthly income		
≤SR 5,000	303	49.2
SR 5,001 – 10,000	69	11.2
SR 10,001 – 15,000	94	15.3
SR 15,001 – 20,000	90	14.6
>SR 20,000	60	9.7

SR, Saudi Riyal (SR 3.75 = $1).

### 3.2 Associations between knowledge, attitudes, and practices toward dengue fever and sociodemographic characteristics among the public in the Western region of Saudi Arabia

The mean score of participants’ knowledge of DF was 20.4 ± 5.57, while the median score was 21.0 (16.0–24.0). Province of residency has been found to be significantly associated with knowledge related to DF (*p* < 0.001). Results show a significantly greater median total knowledge score was found among the residents of Makkah province as compared to Madinah province (*p* < 0.001). The different age group, gender and marital status of participants show no significant associations with total knowledge score. Participants’ total knowledge of DF has been found to be significantly associated with educational level (*p* < 0.001). Results of pairwise comparisons indicate a significantly higher average knowledge score among participants who reported having postgraduate degree compared to < high-school, high-school and diploma (*p* = 0.001, *p* = 0.009 and *p* = 0.002, respectively). The median total score of participants’ knowledge was significantly linked to employment status (*p* < 0.001). Pairwise comparisons show that a significantly superior median knowledge score was found among participants who reported working in health sectors as compared to participants who reported being unemployed or working in non-health sectors (*p* < 0.001 and *p* = 0.002), respectively. Participants’ knowledge of DF has been found to be significantly associated with monthly income (*p* = 0.031). Pairwise comparisons indicate a significantly higher knowledge of DF among participants who reported a monthly income of > SR 20,000 as compared to SR 5,001–10,000 (*p* = 0.015).

The mean score of participants’ attitudes toward DF was 4.11 ± 1.27, while the median score was 4.00 (3.00–5.00). Participants’ attitude toward DF was not significantly associated with participants’ province of residency (*p* = 0.051). Participants’ attitude toward DF was significantly associated with participants age (*p* < 0.001). Pairwise comparisons show significantly greater average attitude score was found among participants aged 31–50 years compared to participants aged 18–30 years (*p* < 0.001) and among participants aged >50 years compared to participants aged 18–30 years (*p* < 0.001). Results show no significant associations in total attitude among different genders of participants. Participants’ attitude toward DF was significantly associated with marital status of participants (*p* < 0.001). In addition, results show a significantly higher average score of married participants than single participants (*p* < 0.001). Participants’ attitude toward DF was not significantly associated with the education level of participants (*p* = 0.063). Participants’ attitude toward DF was significantly associated with employment status (*p* < 0.001). Pairwise comparisons show a significantly higher average total attitude score among participants who reported being unemployed, employed in non-health sectors or employed in health sectors compared to students (*p* = 0.009, *p* < 0.001 and *p* = 0.038, respectively). The average attitude score of participants was significantly associated with monthly income (*p* < 0.001). Pairwise comparisons indicate that a significantly higher average total attitude score was found among participants who reported a monthly income of > SR 20,000 and SR 10,001–15,000 as compared to participants who reported a monthly income of < SR 5,000 (*p* < 0.001, for both).

The mean score of participants’ practices toward DF was 2.84 ± 2.58, while the median score was 3.0 (0.00–5.00). Participants’ practices toward preventing DF was significantly associated with the province of residence (*p* < 0.001). In addition, results show a significantly higher median total practice score among Makkah province participants than Madinah province participants (*p* < 0.001). Participants’ practices toward preventing DF was significantly related with age of participants (*p* < 0.001). Pairwise comparisons show significantly greater practices related to the prevention of DF were found among participants aged 31–50 years and > 50 years as compared to participants aged 18–30 years (*p* = 0.032 and *p* < 0.001). Furthermore, there was no significant relations in total practice among different genders of participants. Participants’ practices toward preventing DF was significantly associated with the marital status of participants (*p* < 0.001). Results show significantly higher median score of married couple participants than single participants (*p* < 0.001). Participants’ practices toward DF prevention were significantly associated with participants’ education levels (*p* = 0.038). Moreover, participants’ practices toward DF prevention were significantly associated with participants’ employment status (*p* = 0.001), where the total score of practices was significantly higher among unemployed participants or employed in non-health sectors as compared to students (*p* = 0.003 and *p* = 0.012), respectively. The average score of participants’ practices toward prevention of DF was significantly associated with monthly income (*p* < 0.001). Pairwise comparisons show that significantly superior practice scores related to prevention of DF were found among participants who reported a monthly income of SR 10,001–15,000 compared to participants who reported a monthly income of < SR 5,000 (*p* < 0.001). Detailed data concerning the association between participants’ KAP toward DF and the features of participants are provided in Table 3.

**Table 3:** Knowledge, attitudes, and practices toward dengue fever (DF) according to participants’ characteristics in the Western region of Saudi Arabia.

Characteristics	DF knowledge score out of 35 Mean ± SD Median (IQR)	DF attitude score out of 5 Mean ± SD Median (IQR)	DF practice score out of 8 Mean ± SD Median (IQR)
Province of residency			
Makkah	20.9 ± 5.47 21.0 (17.0–25.0)	4.15 ± 1.26 4.00 (3.00–5.00)	3.04 ± 2.55 3.00 (0.00–5.00)
Madinah	17.6 ± 5.27 18.0 (14.0–21.0)	3.90 ± 1.31 4.00 (3.00–5.00)	1.80 ± 2.51 0.00 (0.00–4.00)
*p*-value	<0.001*	0.051	<0.001*
Age in years			
18–30	20.6 ± 5.75 22.0 (16.0–24.5)	3.73 ± 1.25 4.00 (3.00–5.00)	2.35 ± 2.44 2.00 (0.00–4.00)
31–50	20.5 ± 5.36 20.0 (17.0–24.0)	4.39 ± 1.16 4.50 (4.00–5.00)	2.97 ± 2.68 3.00 (0.00–5.00)
>50	19.6 ± 5.46 19.0 (15.0–23.0)	4.56 ± 1.25 5.00 (4.00–6.00)	3.78 ± 2.47 4.00 (2.00–6.00)
*p*-value	0.141	<0.001*	<0.001*
Gender			
Male	20.14 ± 5.92 21.0 (16.0–24.0)	4.13 ± 1.34 4.00 (3.00–5.00)	2.93 ± 2.59 3.00 (0.00–5.00)
Female	20.71 ± 4.98 21.0 (17.0–24.0)	4.10 ± 1.18 4.00 (3.00–5.00)	2.71 ± 2.56 3.00 (0.00–5.00)
*p*-value	0.253	0.545	0.339
Marital status			
Single	20.71 ± 5.65 21.0 (17.0–25.0)	3.83 ± 1.25 4.00 (3.00–5.00)	2.47 ± 2.47 2.00 (0.00–5.00)
Married	20.02 ± 5.48 20.0 (16.0–24.0)	4.41 ± 1.23 4.00 (3.00–5.00)	3.22 ± 2.63 4.00 (0.00–5.00)
*p*-value	0.054	<0.001*	<0.001*
Education level			
<High-school	15.17 ± 4.30 14.0 (11.0–18.8)	3.58 ± 1.44 3.00 (3.00–5.00)	1.42 ± 1.78 0.50 (0.00–2.75)
High-school	19.3 ± 5.39 20.0 (15.0–24.0)	4.01 ± 1.30 4.00 (3.00–5.00)	2.51 ± 2.58 2.00 (0.00–5.00)
Diploma	17.83 ± 5.46 16.0 (14.0–22.3)	4.53 ± 1.07 4.00 (4.00–6.00)	3.50 ± 2.42 4.00 (0.75–5.25)
Bachelor	20.5 ± 5.53 21.0 (16.0–24.0)	4.06 ± 1.29 4.00 (3.00–5.00)	2.80 ± 2.51 3.00 (0.00–5.00)
Postgraduate	22.1 ± 5.28 22.0 (19.0–26.0)	4.35 ± 1.20 4.00 (3.00–5.00)	3.30 ± 2.82 4.00 (0.00–6.00)
*p*-value	<0.001*	0.063	0.038*
Employment status			
Unemployed	18.71 ± 5.30 19.0 (15.0–22.0)	4.18 ± 1.31 4.00 (3.00–5.00)	3.26 ± 2.55 4.00 (0.00–5.00)
Student	21.1 ± 5.55 22.0 (17.0–25.0)	3.76 ± 1.22 4.00 (3.00–5.00)	2.34 ± 2.38 2.00 (0.00–4.00)
Employed in non-health sector	20.0 ± 5.32 20.0 (16.0–24.0)	4.42 ± 1.24 5.00 (4.00–5.00)	3.19 ± 2.73 3.00 (0.00–6.00)
Employed in health sector	22.9 ± 5.65 23.0 (19.0–28.0)	4.27 ± 1.20 4.00 (3.00–5.00)	2.49 ± 2.56 2.00 (0.00–5.00)
*p*-value	<0.001*	<0.001*	0.001*
Monthly income			
≤ SR 5,000	20.3 ± 5.59 21.0 (16.0–24.0)	3.83 ± 1.24 4.00 (3.00–5.00)	2.48 ± 2.45 2.00 (0.00–4.00)
SR 5,001 – 10,000	18.8 ± 5.48 19.0 (14.0–23.0)	4.19 ± 1.28 4.00 (3.00–5.00)	2.83 ± 2.85 2.00 (0.00–6.00)
SR 10,001 – 15,000	20.8 ± 4.94 21.0 (17.0–24.3)	4.72 ± 1.07 5.00 (4.00–6.00)	3.87 ± 2.51 4.00 (1.75–6.00)
SR 15,001 – 20,000	20.4 ± 5.37 21.0 (16.00–24.0)	4.09 ± 1.30 4.00 (3.00–5.00)	2.87 ± 2.50 3.00 (0.00–5.00)
> SR 20,000	22.0 ± 6.36 22.0 (18.0–28.0)	4.57 ± 1.28 5.00 (4.00–6.00)	3.03 ± 2.74 3.00 (0.00–5.75)
*p*-value	0.031*	<0.001*	<0.001*

*Alpha was set at 0.05 to denote significance. SR, Saudi Riyal (SR 3.75 = $1); SD, standard deviation; IQR, interquartile range.

### 3.3 Predictors of knowledge, attitudes, and practices regarding dengue fever among the public in the Western region of Saudi Arabia

The results of the linear regression analyses between sociodemographic features and participants’ knowledge toward DF show that participants’ province of residency predicted participants’ knowledge negatively, in which being a residence of Madinah predicted lower participants’ knowledge [B: -3.30, SE: 0.60 (95% CI: −4.47 to −2.13), *p* < 0.001]. Higher participants’ education level predicted participants’ knowledge positively [B: 1.10, SE: 0.22 (95% CI: 0.66 to 1.52), *p* < 0.001]. In addition, participants’ employment status predicted participants’ knowledge positively, where being employed in health sectors predicted higher participants’ knowledge [B: 0.99, SE: 0.23 (95% CI: 0.54 to 1.44), *p* < 0.001].

The results of the linear regression analyses between sociodemographic features and participants’ attitude toward DF show that higher participants’ age and monthly income predicted participants’ attitude positively [B: 0.45, SE: 0.06 (95% CI: 0.33 to 0.58), *p* < 0.001] and [B: 0.18, SE: 0.04 (95% CI: 0.11 to 0.25), *p* < 0.001], respectively. Moreover, participants’ marital status predicted participants’ attitude positively, in which being married predicted higher participants’ attitude [B: 0.58, SE: 0.10 (95% CI: 0.38 to 0.78), *p* < 0.001]. Participants’ employment status predicted participants’ attitude positively, where being employed in non-health sectors predicted higher participants’ attitude [B: 0.11, SE: 0.05 (95% CI: 0.01 to 0.22), *p* = 0.034].

The results of the linear regression analyses between sociodemographic features and participants’ practices toward DF show that participants’ province of residency predicted participants’ practices negatively, in which being a residence of Madinah predicted lower participants’ preventive practices against DF [B: -1.25, SE: 0.28 (95% CI: −1.80 to −0.70), *p* < 0.001]. Moreover, participants’ marital status predicted participants’ practices positively, in which being married predicted higher participants practices [B: 0.74, SE: 0.21 (95% CI: 0.34 to 1.15), *p* < 0.001]. In addition, higher participants’ age, education level and monthly income predicted participants’ practices positively [B: 0.70, SE: 0.13 (95% CI: 0.44 to 0.96), *p* < 0.001], [B: 0.24, SE: 0.10 (95% CI: 0.04 to 0.45), *p* = 0.020] and, [B: 0.20, SE: 0.07 (95% CI: 0.05 to 0.34), *p* = 0.007], respectively. Results obtained from the linear regression analyses of predictors of participants’ KAP toward DF are provided in Table 4.

**Table 4:** Linear regression analysis of predictors of knowledge, attitudes, and practices regarding dengue fever (DF) among the public in the Western region of Saudi Arabia.

	Beta	Standard error	95% Confidence interval	*p*-value	R-square
Knowledge of participants toward DF					
Province of residency	–3.30	0.60	−4.47 – 2.13	<0.001*	0.05
Age in years	−0.48	0.29	−1.04 – 1.00	0.106	0.00
Gender	0.57	0.46	−0.33 – 1.47	0.216	0.00
Marital status	−0.69	0.45	−1.57 – 0.19	0.123	0.00
Education level	1.10	0.22	0.66 – 1.52	<0.001*	0.04
Employment status	0.99	0.23	0.54 – 1.44	<0.001*	0.03
Monthly income	0.29	0.16	−0.02 – 0.60	0.066	0.01
Attitude of participants toward DF					
Province of residency	−0.27	0.14	−0.53 – 0.02	0.067	0.01
Age in years	0.45	0.06	0.33 – 0.58	<0.001*	0.08
Gender	−0.03	0.11	−0.24 – 0.18	0.772	0.00
Marital status	0.58	0.10	0.38 – 0.78	<0.001*	0.05
Education level	0.09	0.05	−0.02 – 0.19	0.095	0.01
Employment status	0.11	0.05	0.01 – 0.22	0.034*	0.01
Monthly income	0.18	0.04	0.11 – 0.25	<0.001*	0.04
Practices of participants toward DF					
Province of residency	−1.25	0.28	−1.80 – 0.70	<0.001*	0.03
Age in years	0.70	0.13	0.44 – 0.96	<0.001*	0.04
Gender	−0.22	0.22	−0.64 – 0.20	0.308	0.00
Marital status	0.74	0.21	0.34 – 1.15	<0.001*	0.02
Education level	0.24	0.10	0.04 – 0.45	0.020*	0.01
Employment status	−0.10	0.11	−0.31 – 0.12	0.372	0.00
Monthly income	0.20	0.07	0.05 – 0.34	0.007*	0.01

*Alpha was set at 0.05 to denote significance.

## 4 Discussion

Dengue fever is a viral infection known to spread to humans by the *Aedes aegypti* kind of mosquitos and it has the potential to cause illness and hospitalization, and in severe cases, it is fatal (2, 3). A review study conducted in Saudi Arabia revealed that a substantial influx of migrant laborers and religious pilgrims influences the dissemination of DF in Saudi Arabia (9). Moreover, the rainy season and elevated humidity levels are critical contributors to the proliferation of mosquito populations, and these factors play significant roles in the transmission of DF (1). The findings of this KAP study showed a good knowledge and attitude toward DF among participants. There was no correlation between participants’ knowledge and attitude. Spearman correlation between participants’ knowledge and practices as well as participants’ attitude and practices were positively low. Moreover, participants’ education level and employment status predicted participants’ knowledge of DF. Participants’ age, marital status, employment status and monthly income predicted participants’ attitude toward DF. Finally, participants’ age, marital status, education level and monthly income predicted participants’ practices toward DF.

Many studies, including a study performed in Jazan, Saudi Arabia, showed a favorable correlation between good knowledge about DF and good socioeconomic status (20), such as educational level (5, 20, 21), employment status (20) and monthly income (5, 22). Aligning with these studies, our findings showed that participants’ education level and employment status predicted participants’ knowledge of DF. This might be because individuals with good socioeconomic status tend to participate more in workplace health initiatives.

The good knowledge related to DF reported among participants in this study has also been reported in other settings. A study conducted in Yemen showed good knowledge of DF. In addition, they reported that more than 90% responded correctly to fever, headache and joint pain as symptoms of DF (13). This finding is consistent with our study, in which most participants reported that fever, headache and joint pain are symptoms of DF. While DF may resolve on its own, some patients may develop serious complications such as dengue hemorrhagic fever and may face death (23). Nevertheless, our analysis showed that fewer participants stated that blood vessel damage is one of the severe complications of DF, and about one-third of participants stated that DF can be fatal. A study conducted in Yemen indicated that more than 84.6% of participants correctly listed the causative agent of DF as a mosquito and not direct contact with an affected person (13). Similarly, our findings demonstrate that most participants indicated mosquito bites as the correct transmission mode, not direct contact with an infected person. The World Health Organization stated that the mosquitos responsible for DF transmission are active during the day (24). Furthermore, a previous study demonstrated that 92% of respondents correctly reported that the mosquitos like to bite in the early morning and late evening (20). In contrast, our findings showed that less than one-third of participants correctly reported that the peak time of mosquito bites is early morning or late afternoon. This dissimilarity may be due to climate and cultural behaviors, in which the high daytime temperature in the WR of Saudi Arabia discourages outdoor gatherings and activities.

This study showed that factors predicted participants’ attitude toward DF included participants’ age, marital status, employment status and monthly income. This result was similar to findings reported in a Malaysian study, in which univariant analysis showed that a good attitude was connected with marital and employment status (20). Similarly, in our study, married couples tend to have a better attitude toward controlling DF than single participants. This can be attributed to the higher sense of responsibility toward their families than single individuals who may depend more on their families. In accordance with our findings, another study concluded that individuals with higher family income had better attitude toward DF prevention (25); this suggests that participants with higher income tend to have increased access to resources, better healthcare, and improved living conditions. Interestingly, our findings indicated that half of the participants reported that DF symptoms overlap with COVID-19 symptoms. This confusion is plausibly linked to the recent outbreak of COVID-19, during which people have become familiar with COVID-19 symptoms, such as fever, headache and general body aches, through media or healthcare providers, which can be easily mistaken for DF clinical manifestations.

In the present study, participants’ age, marital status, education level and monthly income predicted positive participants’ practices toward DF prevention. Even though healthcare professionals demonstrated better knowledge of DF than participants working in non-health sectors, there was no significant positive practices toward preventing DF. Similar results were found by a study in Ecuador, which determined that participants working in health sectors had unsatisfactory practices toward DF prevention (26). The good knowledge about DF among healthcare professionals may be due to the fact that they have easier access to reliable information. However, national health organizations and stakeholders should provide educational campaigns among health sectors to raise their practices toward DF prevention.

In addition, in the past few years, the heavy rainfalls have increased remarkably in the WR of Saudi Arabia. A previous study in Makkah showed that the heavy rainfalls were followed by increased DF incidence (18), possibly due to climate change and the water stagnation following such heavy rainfalls. Nowadays, particularly post-COVID-19 pandemic, there has been a notable surge in internet exposure and utilization of digital platforms. The Ministry of Municipal and Rural Affairs and Housing in Saudi Arabia has developed a platform called “Balady,” through which people can report stagnant water, as a service that encourages the idea of community collaboration to enhance the quality of life and services offered to recipients (27). In this study, the majority of participants believed that society should work together to prevent DF. Moreover, two-thirds of participants felt responsible for reporting stagnant water to avoid DF infection; however, approximately half of them did not know how to report. Our finding also showed that the most quoted source of information about DF is the internet and social media (57.8%), which aligns with the conclusion drawn by Purnama and colleagues, who found that social media is the foremost source of information as compared to other electronic sources (28), suggesting that participants allocate significant time to the internet and social media. This discrepancy between the considerable time spent on the internet and the unawareness about the Balady platform may be attributed to the lack of effective marketing strategies and educational efforts about the platform through the internet and social media. Therefore, raising awareness about DF prevention and available platforms through the internet and social media may contribute to reducing the incidence of DF among the WR public of Saudi Arabia.

To the best of our knowledge, this is the first study conducted on DF among the WR public of Saudi Arabia. However, this study might be limited by its cross-sectional design, where change in knowledge and how it can affect practices related to DF cannot be assessed. The generalizability of the study results also might be limited due to the convenient sampling method used to recruit participants. In fact, residents of Saudi Arabia widely use social media applications, and almost everyone has constant internet access.

## 5 Conclusion

Western region KAP toward DF can be predicted by sociodemographic traits. Urgent intervention to enhance public practices for DF prevention is needed. Nationwide longitudinal studies should investigate the causal relationship between public and practices toward preventing DF and sociodemographic characteristics in the future.

## Data availability statement

The raw data supporting the conclusions of this article will be made available by the authors, without undue reservation.

## Ethics statement

The studies involving humans were approved by the Biomedical Research Ethics Committee at Umm Al-Qura University [certification No. HAPO-02-K-012-2023-06-1642]. The studies were conducted in accordance with the local legislation and institutional requirements. The participants provided their written informed consent to participate in this study.

## Author contributions

MH: Conceptualization, Data curation, Formal analysis, Investigation, Methodology, Project administration, Resources, Writing – original draft, Writing – review & editing.

## References

[1] Simmons CP Farrar JJ van Vinh CN Wills B. Dengue. N Engl J Med. (2012) 366:1423–32. doi: 10.1056/NEJMra1110265 22494122

[2] Bhatt S Gething PW Brady OJ Messina JP Farlow AW Moyes CL. The global distribution and burden of dengue. Nature. (2013) 496:504–7. doi: 10.1038/NATURE12060, PMID: 23563266 PMC3651993

[3] Wilder-Smith A Gubler DJ Weaver SC Monath TP Heymann DL Scott TW. Epidemic arboviral diseases: priorities for research and public health. Lancet Infect Dis. (2017) 17:e101–6. doi: 10.1016/S1473-3099(16)30518-7, PMID: 28011234

[4] Gubler DJ. Dengue, urbanization and globalization: the unholy trinity of the 21st century. Trop Med Health. (2011) 39:S3–S11. doi: 10.2149/TMH.2011-S05, PMID: 22500131 PMC3317603

[5] Ahmed AE Almarhabi MA Shami MO Alhazemi AA Alsharif HM Abu Hayyah AE. Knowledge, attitudes, and practices of the population in Jazan region, Saudi Arabia regarding dengue fever and its prevention measures: a community-based cross-sectional study. Int J Environ Res Public Health. (2022) 19:812. doi: 10.3390/IJERPH192416812, PMID: 36554693 PMC9779683

[6] Brady OJ Gething PW Bhatt S Messina JP Brownstein JS Hoen AG. Refining the global spatial limits of dengue virus transmission by evidence-based consensus. PLoS Negl Trop Dis. (2012) 6:e1760. doi: 10.1371/JOURNAL.PNTD.0001760, PMID: 22880140 PMC3413714

[7] Al-Raddadi R Alwafi O Shabouni O Akbar N Alkhalawi M Ibrahim A. Seroprevalence of dengue fever and the associated sociodemographic, clinical, and environmental factors in Makkah, Madinah, Jeddah, and Jizan, Kingdom of Saudi Arabia. Acta Trop. (2019) 189:54–64. doi: 10.1016/J.ACTATROPICA.2018.09.009, PMID: 30244133

[8] Damtew YT Tong M Varghese BM Anikeeva O Hansen A Dear K. Effects of high temperatures and heatwaves on dengue fever: a systematic review and meta-analysis. EBioMedicine. (2023) 91:104582. doi: 10.1016/j.ebiom.2023.104582, PMID: 37088034 PMC10149186

[9] Altassan KK Morin C Shocket MS Ebi K Hess J. Dengue fever in Saudi Arabia: a review of environmental and population factors impacting emergence and spread. Travel Med Infect Dis. (2019) 30:46–53. doi: 10.1016/J.TMAID.2019.04.006 30978417

[10] Guzman MG Harris E. Dengue. Lancet. (2015) 385:453–65. doi: 10.1016/S0140-6736(14)60572-9 25230594

[11] Hadinegoro SR. The revised WHO dengue case classification: does the system need to be modified? Paediatr int child. Health. (2012) 32:33–8. doi: 10.1179/2046904712Z.00000000052, PMID: 22668448 PMC3381438

[12] Wang WH Urbina AN Chang MR Assavalapsakul W Lu PL Chen YH. Dengue hemorrhagic fever – a systemic literature review of current perspectives on pathogenesis, prevention and control. J Microbiol Immunol Infect. (2020) 53:963–78. doi: 10.1016/J.JMII.2020.03.007 32265181

[13] Alyousefi TAA Abdul-Ghani R Mahdy MAK Al-Eryani SMA Al-Mekhlafi AM Raja YA. A household-based survey of knowledge, attitudes and practices towards dengue fever among local urban communities in Taiz governorate, Yemen. BMC Infect Dis. (2016) 16:1–9. doi: 10.1186/S12879-016-1895-2/TABLES/5 27717333 PMC5054547

[14] Van NH Than PQT Nguyen TH Vu GT Hoang CL Tran TT. Knowledge, attitude and practice about dengue fever among patients experiencing the 2017 outbreak in Vietnam. Int J Environ Res Public Health. (2019) 16:976. doi: 10.3390/IJERPH16060976, PMID: 30889912 PMC6466316

[15] Al-Zurfi BMN Fuad M Abdelqader MA Baobaid M Elnajeh M Ghazi HF. Knowledge, attitude and practice of dengue fever and heath education programme among students of Alam Shah science school, Cheras, Malaysia. Malays J Public Health Med. (2015) 2015:69–74.

[16] Elsinga J Lizarazo EF Vincenti MF Schmidt M Velasco-Salas ZI Arias L. Health seeking behaviour and treatment intentions of dengue and fever: a household survey of children and adults in Venezuela. PLoS Negl Trop Dis. (2015) 9:e0004237. doi: 10.1371/JOURNAL.PNTD.0004237, PMID: 26624283 PMC4666462

[17] Ruberto I Yaglom H Erhart LM Plante L Weiss J Golenko C. Dengue knowledge, attitudes, and practices among Arizona health care providers, 2014–2015. Vector Borne Zoonotic Dis. (2019) 19:434–40. doi: 10.1089/VBZ.2018.2370 30802177

[18] Melebari S Bakri R Hafiz A Qabbani F Khogeer A Alharthi I. The epidemiology and incidence of dengue in Makkah, Saudi Arabia, during 2017-2019. Saudi Med J. (2021) 42:1173–9. doi: 10.15537/SMJ.2021.42.11.20210124, PMID: 34732548 PMC9149740

[19] Saghir MA Ahmed WAM Dhaiban MMA Osman ME Abduljabbar NI. Knowledge, attitude, and practices of the community toward dengue fever in Shabwah governorate, Yemen: a descriptive study. J Egypt Public Health Assoc. (2022) 97:1–8. doi: 10.1186/S42506-022-00121-5/TABLES/7 36464718 PMC9719877

[20] Selvarajoo S Liew JWK Tan W Lim XY Refai WF Zaki RA. Knowledge, attitude and practice on dengue prevention and dengue seroprevalence in a dengue hotspot in Malaysia: a cross-sectional study. Sci Rep. (2020) 10:1–13. doi: 10.1038/s41598-020-66212-5, PMID: 32533017 PMC7293214

[21] Wong LP Shakir SMM Atefi N AbuBakar S. Factors affecting dengue prevention practices: Nationwide survey of the Malaysian public. PLoS One. (2015) 10:e0122890. doi: 10.1371/JOURNAL.PONE.0122890, PMID: 25836366 PMC4383514

[22] Phuyal P Kramer IM Kuch U Magdeburg A Groneberg DA Lamichhane Dhimal M. The knowledge, attitude and practice of community people on dengue fever in Central Nepal: a cross-sectional study. BMC Infect Dis. (2022) 22:1–18. doi: 10.1186/S12879-022-07404-4/TABLES/6 35549884 PMC9096776

[23] Ho TS Huang MC Wang SM Hsu HC Liu CC. Knowledge, attitude, and practice of dengue disease among healthcare professionals in southern Taiwan. J Formos Med Assoc. (2013) 112:18–23. doi: 10.1016/J.JFMA.2012.11.004, PMID: 23332425

[24] Dengue and Severe Dengue. Available at: https://www.who.int/en/news-room/fact-sheets/detail/dengue-and-severe-dengue (Accessed October 24, 2023).

[25] Ghani NA Shohaimi S Hee AKW Chee HY Emmanuel O Ajibola LSA. Comparison of knowledge, attitude, and practice among communities living in hotspot and non-hotspot areas of dengue in Selangor, Malaysia. Trop Med Infect Dis. (2019) 4:37. doi: 10.3390/TROPICALMED4010037 30781369 PMC6473475

[26] Handel AS Ayala EB Borbor-Cordova MJ Fessler AG Finkelstein JL Espinoza RXR. Knowledge, attitudes, and practices regarding dengue infection among public sector healthcare providers in Machala, Ecuador. Trop Dis Travel Med Vaccines. (2015) 2:1–10. doi: 10.1186/S40794-016-0024-Y/TABLES/7 PMC5531027 28883952

[27] About Balady. *Balady platform*. Available at: https://balady.gov.sa/en/about-balady (Accessed October 20, 2023).

[28] Purnama SG Susanna D Achmadi UF Eryando T. Attitude towards dengue control efforts with the potential of digital technology during COVID-19: partial least squares-structural equation modeling. F1000Res. (2023) 11:1283. doi: 10.12688/f1000research.125318.2 37441548 PMC10333779

